# Heat stress during the milky stage reshapes phenology, assimilate partitioning, and yield formation in rice cultivars with contrasting heat tolerance

**DOI:** 10.3389/fpls.2026.1875326

**Published:** 2026-07-09

**Authors:** Payu Pansarakham, Netnapa Sumtonglang, Panupon Hongpakdee, Charanya Kulya, Piyada Theerakulpisut, Anoma Dongsansuk

**Affiliations:** 1 Research Center and Central Laboratory, Faculty of Agriculture, Khon Kaen University, Khon Kaen, Thailand; 2 Department of Agronomy, Faculty of Agriculture, Khon Kaen University, Khon Kaen, Thailand; 3 Salt-Tolerant Rice Research Group, Khon Kaen University, Khon Kaen, Thailand; 4 Department of Horticulture, Faculty of Agriculture, Khon Kaen University, Khon Kaen, Thailand; 5 Department of Science and Technology, Faculty of Liberal Arts and Science, Roi-Et Rajabhat University, Roi Et, Thailand; 6 Department of Biology, Faculty of Science, Khon Kaen University, Khon Kaen, Thailand

**Keywords:** heat stress, phenology, photo-assimilate translocation, rice, yield

## Abstract

Heat stress during the grain-filling period of rice cultivars leads to a reduction in both yield quality and quantity. This research examined the effect of milky-stage heat stress (milky HT; 41/28°C day/night, for 7 days) on phenology, partitioning, and yield in 12 rice cultivars compared to standard heat-tolerant (N22 and Dular) and heat-sensitive (IR64) cultivars. Milky HT caused a shortened phenology from milky to physiological maturity in RD41, N22, and Dular, whereas others exhibited prolonged phenology. Milky HT influenced partitioning, with RD41 increasing dry weight in all parts, and with PSL2 rapidly assimilating translocation to grains. However, CN1 and RD49 experienced rapid dry weight loss across all parts. Additionally, milky HT caused a significant reduction in all yield components and overall yield in RD63, while N22 maintained its parameters. Phenology shift (from milky to physiological maturity and from dough to physiological maturity) and seed set were considered key indicators associated with heat tolerance. Based on these traits, rice cultivars were classified into four groups: heat-tolerant (N22, Dular, and SPT1), moderate heat-tolerant (PSL2, RD61, Rice berry, and RD41), moderate heat-sensitive (CN1), and heat-sensitive (IR64, PTT1, RD29, RD31, RD49, RD57, and RD63). These findings revealed that heat-tolerant rice under milky HT is associated with different phenological and assimilate allocation strategies, in addition to clarifying useful traits and rice seed resources for a heat-tolerance breeding program.

## 1 Introduction

Rice is a staple food source and an essential economic crop in Thailand. It is cultivated across the country throughout the year, relying on rainfall during the rainy season and irrigation during the drier months. As the 21^st^ century progresses, air temperature is estimated to rise by 1.5 °C–4.5°C due to global warming (Peraudeau et al., 2015). Thus, plant heat stress is linked to climate change and extreme weather events. Rising and elevated temperatures (Rani and Maragatham, 2013; Kumar et al., 2021) induce heat stress in rice (Shrestha et al., 2022; Chandarak et al., 2023), significantly impacting rice production (Zhang, 2022) by disrupting growth and development, interfering with plant metabolism and physiological mechanisms (Xu et al., 2021), and reducing rice grain filling (Ali et al., 2019).

Crops, including rice, typically exhibit efficient physiological mechanisms, growth, reproduction, and yield potential within an optimal temperature range. However, their mechanisms can be affected above or below optimum temperature ranges, causing potential yield loss (Shrestha et al., 2022). Currently, elevated environmental temperatures cause heat stress, which influences rice growth at various growth stages, such as vegetative (Daubenmire, 1974), reproductive, and grain-filling stages, depending on cultivars and season. In particular, the reproductive stage is considered a crisis growth stage under heat stress (Satake and Yoshida, 1978). Heat stress can change rice phenology (Oort et al., 2011; Zhang and Tao, 2013 and Sanwong et al., 2023) by shortening or prolonging phenology shift (Zhang, 2022). In Indian rice, key growth stages, including tillering, panicle initiation, flowering, and harvesting stages, are impacted by heat stress, ultimately reducing the phenology at the harvesting period (Rani and Maragatham, 2013).

Phenology indicates the growth duration of crops, along with ambient temperature, rainfall, and sunlight duration (Chen and Gong, 2021). Among these factors, phenology and ambient temperature play a crucial role in determining growing degree days (GDD), as they are directly interconnected and influence each other (Zhang et al., 2022). Studies have found that GDD during transplanting and tillering stages is significantly correlated with grain yield (Medhi et al., 2019). In addition, accumulated growing degree days (AGDD) also cause an impact on rice phenology shift. An increase in AGDD reduces rice phenology during anthesis and embryo and endosperm development, leading to reduced embryo size, slower embryo growth rate, and ultimately rice yield reduction (Sanwong et al., 2023).

Heat stress affects assimilate translocation, partitioning, and food storage within different parts of rice plants, ultimately leading to rice yield reduction (Shi et al., 2015b; Xu et al., 2021). It also disrupts physiological mechanisms. High night temperature stress affects rice at the early milky stage by disrupting the electron transport in the mitochondria. This leads to H^+^ concentration changes in mitochondria and affects enzyme activity in the Krebs cycle (Liao et al., 2015). Heat stress also impairs thylakoid membrane permeability and decreases chlorophyll content, resulting in altered photochemistry, decreased chlorophyll fluorescence, and reduced net photosynthetic rate (Xu et al., 2021). It also induces markedly increased intracellular reactive oxygen species (ROS), leading to impairment of membrane structure and function and increasing electrolyte leakage (Xu et al., 2021), ultimately reducing yield. In a previous study, Lü et al. (2013) reported that the risk of heat stress on rice at flowering and grain-filling stages exhibited no reduced pattern in net photosynthetic rate. Meanwhile, dry weight in leaves, leaf sheaths, culms, and panicles decreased, and grain yield decreased by 15%–73%. A recent study also demonstrated that heat stress severely affects plant growth and yield through physiological and molecular mechanisms, such as damaged photosynthetic performance, changed carbohydrate metabolism, induced senescence, and decreased grain-filling potential. These responses caused a decline in grain yield and quality, especially where heat stress occurred during reproductive and grain-filling stages (Kumar et al., 2025). In addition, heat stress reduced dry matter accumulation in leaves and flowers during the flowering and grain-filling stages, causing lower grain weight (Lü et al., 2013). It also affected key yield components and yield, including tillers, panicles, filled and unfilled grains, 1000-grain weight, yield, and harvest index in rice. Kumar et al. (2021) reported that grain weight tends to decline under heat stress because of a reduction in grain growth rate and duration. At the grain-filling stage, heat stress disrupted regulation of gene encoding starch synthesis, suppressing starch accumulation in rice grains (Xu et al., 2021) and causing an increase in chalky grain (Abayawickrama et al., 2017; Xu et al., 2021). Moreover, exposure to heat stress at 33°C during daytime significantly deteriorated milling quality and increased chalkiness in grains (Abayawickrama et al., 2017).

Rice growth responds differently to heat stress at various growth stages. The sensitivity of rice growth stages to heat stress, from most to least sensitive, is heading and flowering > panicle initiation > grain-filling stages. Heat stress occurring during the reproductive stage (heading and flowering stages) causes shortened pollen germination rate and declining pollen viability, resulting in sterility in rice grains (Zhang, 2022). Ashish et al. (2017) found that rice at flowering and grain-filling stages (early grain filling) was sensitive to heat stress, resulting in yield loss. Thus, the majority of studies have focused on the grain-filling stage (dough and maturity) to understand the impacts of heat stress on rice grain weight. However, heat stress did not show a significant effect on the length and width of rice grains because rice glumes contribute to the final size at the anthesis stage. This impacts grain quantity (Wu et al., 2022). In addition, heat stress occurring at the grain-filling stage causes rapid photosynthate translocation and reduces grain-filling stage duration (post-anthesis), grain weight, and leads to unfilled grains (Zhang, 2022). This affects grain quality. Therefore, developing heat tolerance in rice during the grain-filling stage is crucial because heat stress at this point severely impacts both grain quality and quantity.

Presently, the Thai government has developed various rice cultivars to withstand environmental stresses, including drought, flooding, and salinity. However, studies on heat-tolerant rice remain limited and unclear. The majority of previous studies focused on rice responses to heat stress during the flowering and grain-filling stages, including dough and maturity, while the milky stage has been less studied. This stage is crucial because it is a period when endosperm remains in liquid form, enhancing nutritional value; heat stress here can significantly impact grain quality and quantity. Rice harvested at this stage is traditionally valued as a nourishing food, as it is believed to be easily digestible and contains higher bioactive compounds compared to the mature stage (Jiamyangyuen et al., 2017; Ngamsuk et al., 2020). The milky stage involves carbon assimilation and final grain yield determination, further justifying the need to determine rice responses to milky-stage heat stress. The novelty of this study is based on identifying key heat-tolerance traits and multivariate classification of Thai rice cultivars depending on their phenology, assimilate partitioning, and yield responses to milky HT. Therefore, this research aims to study the assimilate translocation, growth duration, yield components, and yield of 12 recommended rice cultivars at the milky stage in response to heat stress, compared with standard rice cultivars such as heat-tolerant (N22 and Dular) and heat-sensitive (IR64). Heat stress treatment involved exposure to 41 °C (3 h/day) for 7 days, simulating the severe temperatures in tropical rice-growing conditions. This temperature was based on the average highest temperature during March–May 2016 and 2017 at the Agronomy Field Station, Faculty of Agriculture, Khon Kaen University, Thailand. Exposure to this temperature for 7 days creates a clear distinction between heat-tolerant and heat-sensitive rice varieties. This study provides fundamental knowledge for the classification of heat tolerance and heat sensitivity among the 12 recommended rice cultivars at the milky stage and serves as valuable data for heat tolerance in breeding programs.

## 2 Materials and methods

### 2.1 Preparation of plant materials

In total, 12 rice (*Oryza sativa* L.) cultivars, namely, CN1, PSL2, PTT1, SPT1, RD29, RD31, RD41, RD49, RD57, RD61, RD63, and Rice berry, were used in this experiment. The heat-tolerant rice cultivars N22 and Dular were included as reference standards (Lang et al., 2015), while IR64 served as a heat-sensitive rice (Coast et al., 2016). Seeds were surface-sterilized with Clorox, thoroughly rinsed with tap water, and soaked in distilled water for 24 h. The seeds were then germinated on moist filter paper. At 7 days after germination, the seedlings were transplanted into plastic pots containing paddy soil. The paddy soil in this study had a sandy texture with a pH of 5.18, organic matter content of 0.487%, total nitrogen (N) of 0.0235%, available phosphorus (P) of 5.64 mg kg^-1^, exchangeable potassium (K) of 37.28 mg kg^-1^, and electrical conductivity (EC) of 0.05 dS m^-1^. Plants were grown in an open greenhouse at the Agronomy Field Station, Faculty of Agriculture, Khon Kaen University (16°28′14.62″ N, 102°48′41.35″ E) from May to October 2018.

All rice cultivars were grown under ambient conditions until they reached the milky stage. The milky stage was identified when the first panicle in a rice hill had developing grains containing a moist, milky endosperm. Cultivars reached the milky stage simultaneously for heat treatment. Planting dates were determined according to the growth duration of each cultivar. The timing of the milky stage was calculated based on the expected harvesting date (**Supplementary Table 1**). Rice cultivars were grown on different dates, reaching the milky stage on the same day, and were treated with heat in a temperature chamber. For each rice cultivar, seven pots were grown, and four pots with uniform growth and development at the milky stage were selected for heat treatment. Then all cultivars were subjected to high-temperature treatment to evaluate the effect of heat stress on phenology, partitioning, yield components, and yield. During the milky stage under greenhouse conditions, the maximum, minimum, and average air temperatures were 32 ± 2 °C, 23 ± 1 °C, and 27 ± 1 °C, respectively. For the heat treatment in the controlled temperature chamber, previous climate records collected during March–May 2016 and 2017 at the Agronomy Field Station, Faculty of Agriculture, Khon Kaen University, Thailand, were used as reference conditions to simulate heat conditions within the chamber. All rice experiments were conducted from May to October 2018.

### 2.2 High-temperature treatment

Rice plants at the milky stage were subjected to heat stress for 7 days in a temperature-controlled chamber (VRV. Crop. Ltd, Thailand). The chamber followed a diurnal temperature cycle in which temperature increased during the daytime, reaching a peak of 41 °C for 3 h (00:00–03:00 pm.), after which it declined. The nighttime temperature was maintained at 28 °C, as shown in **Table 1**. After completion of heat treatment, rice plants were returned to the open greenhouse and grown under ambient conditions until the physiological maturity (PM) stage for harvesting. Throughout the experimental period, climate data, including air temperature (T), relative humidity (RH), and light intensity in terms of photosynthetically active radiation (PAR), were recorded using a weather station (WatchDog 1000 Series Micro Stations, Spectrum Technology Inc., Illinois, USA). Air vapor pressure deficit (VPD_air_) was calculated from recorded air temperature and relative humidity data according to Alduchov and Eskridge (1996), using Equation 1. Alteration of VPD affected plant health; high VPD induced plant stress due to increased transpiration.

**Table 1:** Diurnal temperature, relative humidity, and light intensity used to simulate heat stress conditions in the temperature chamber during milky-stage heat stress treatment for 7 days. The maximum temperature of 41°C was maintained for 3 h/day (0:00-3:00 pm), whereas the temperature from 9:00 pm to 03:00 am was maintained at 28°C.

Duration (h)	Temperature (°C)	Relative humidity (%)	Light intensity* (µmol.m^-2^.s^-1^)
00:00–03:00 am	28	65	–
03:00–06:00 am	25	70	–
06:00–07:00 am	25	70	70
07:00–08:00 am	30	65	115
08:00–09:00 am	30	65	200
09:00–10:00 am	35	56	265
10:00–11:00 am	38	45	340
11:00 am–00:00 pm	38	45	390
00:00–01:00 pm	41	40	390
01:00–02:00 pm	41	40	340
02:00–03:00 pm	41	40	265
03:00–04:00 pm	38	45	200
04:00–05:00 pm	38	45	115
05:00–06:00 pm	35	50	70
06:00–09:00 pm	32	55	–
09:00 pm–00:00 am	28	65	–

The air temperature and relative humidity data recorded during March – May 2016 and 2017 at Agronomy field station, Faculty of Agriculture, Khon Kaen University, Thailand, were used as reference conditions for defining the high temperature treatment. Light intensity values (indicated by *) were measured at canopy level, corresponding to the height of rice leaves positioned at 30 cm below the light source.

(1) VPD (kPa) = [0.61078×((1–RH)/100)]×[(17.27 × T)/(T + 237.3)]

where T is the air temperature (°C).

The air temperature and relative humidity data recorded during March–May 2016 and 2017 at the Agronomy Field Station, Faculty of Agriculture, Khon Kaen University, Thailand, were used as reference conditions for defining the high-temperature treatment. Light intensity values (indicated by *) were measured at the canopy level, corresponding to the height of rice leaves positioned 30 cm below the light source.

### 2.3 AGDD calculation

Accumulated growing degree days (AGDD) were represented in terms of the accumulation of temperature and calculated as the cumulative sum of daily growing degree days (GDD). Daily GDD was calculated from recorded air temperatures after milky HT according to McMaster and Wilhelm (1997), as described in (Equation 2):

(2) $$\text{GDD}=\left[\left({\text{T}}_{\text{max}}+{\text{ T}}_{\text{min}}\right)/2\right]-T_{\text{base}}$$

where T_max_, T_min_, and T_base_ are the daily maximum temperature, daily minimum temperature, and base temperature (10°C), respectively.

### 2.4 Determination of phenology

Rice phenological development was recorded throughout the experimental period by daily visual observation at the milky grain, dough grain, and physiological maturity (PM) stages. Daily visual observation followed the standard evaluation system for rice described by Redona (2013). Shortened or prolonged phenology of rice following heat treatment at the milky stage was determined by comparison with their controls.

### 2.5 Determination of dry matter partitioning

At the PM stage, plants were harvested and separated into leaves, stems, roots, and seeds. All samples were dried in a hot-air oven at 80°C to constant weight. Dry weight was recorded for each organ, and dry matter partitioning was expressed as the distribution of organ-specific dry weight. Total dry weight was calculated as the sum of the dry weights of all organs.

### 2.6 Determination of yield components and yield

Rice yield components were assessed at the PM stage, including the number of grains per panicle (GPN), number of filled grains per panicle (FGPN), number of unfilled grains per panicle (UFGPN), 1000-grain weight (1000-GW), and seed set (SS).

### 2.7 Statistical analysis

The experiment was designed as a completely randomized design (CRD) with four biological replications. Each replication consisted of one pot containing one rice plant, and each pot was considered as one experimental unit. Therefore, a total of four plants/four pots (n = 4) were used for each cultivar. Differences among rice cultivars were analyzed by one-way analysis of variance (one-way ANOVA) and Duncan’s Multiple Range Test (DMRT). Comparisons between the control (open greenhouse) and milky-stage heat stress treatments within each rice cultivar were performed using an independent-samples t-test. The relationships among all parameters were evaluated using Pearson’s correlation. All traits were standardized to Z scores before calculating Euclidean distance and performing Ward’s hierarchical clustering. Ward’s hierarchical cluster analysis was performed to classify rice cultivars according to their level of heat tolerance at the milky-stage. All statistical analyses were conducted using SPSS software, version 26.0 (SPSS, Chicago, IL, USA), except for cluster analysis, which was conducted using PC-ORD, version 5.

## 3 Results

### 3.1 Climate conditions during the rice growing period

**Figure 1:**
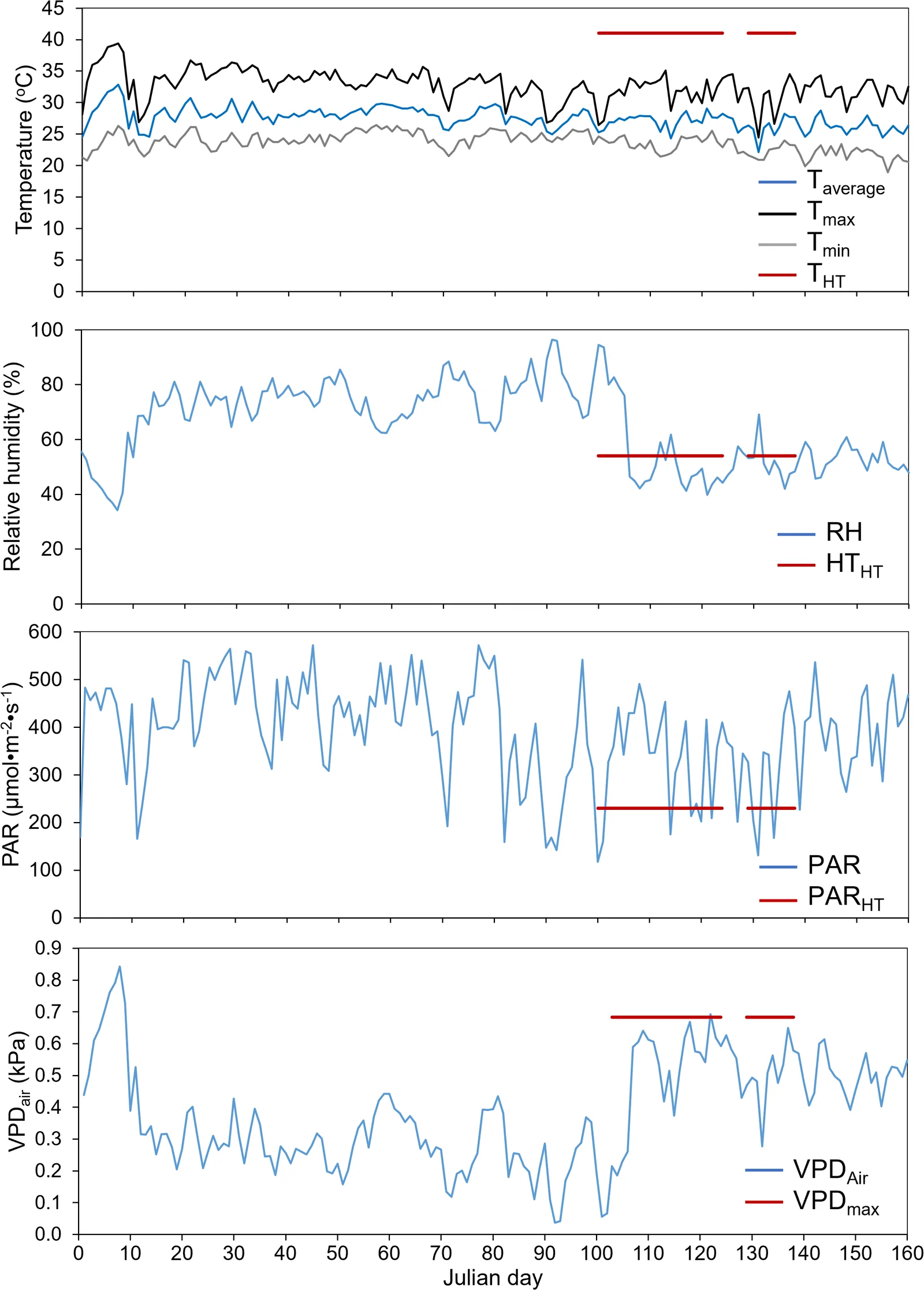
Climate conditions in the open greenhouse and during milky-stage heat stress throughout the rice growing period. The average temperature (T_avr_), maximum temperature (T_max_), minimum temperature (T_min_), relative humidity (RH), light intensity (PAR), and air vapor pressure deficit (VPD_air_) were recorded at the Agronomy Field Station, Khon Kaen University, Thailand, from 17 April to 24 October 2018. .

Climate conditions in the open greenhouse during the experimental period are shown in **Figures 1A–D**. Prior to exposure of milky HT, the overall climate conditions in the open greenhouse were relatively stable, with an average T_avr_ of 27.5°C, T_max_ of 32.7°C, T_min_ of 23.7°C, RH of 65.3%, PAR of 288.4 µmol.m^-2^.s^-1^, and VPD_air_ of 0.38 kPa. The milky HT followed a diurnal temperature cycle (**Table 1**), with a peak temperature of 41 °C maintained for approximately 3 h day^−1^ (00:00–03:00 pm.). During the milky HT treatment, the daily mean daytime temperature (06:00 am–06:00 pm.) was 35.83°C, while the nighttime temperature (06:00 pm–06:00 am) was 28.25°C. This daytime temperature was higher than the T_max_ (31.5°C, **Figure 1A**) recorded in the open greenhouse. In addition, the daytime temperature period was based on ambient air temperature from rice field climate data recorded during March–May 2016 and 2017 at the Agronomy Field Station, Khon Kaen University, where the maximum temperature reached 41°C during the summer season. In contrast, RH during the milky HT period (54%) was similar to that in the open greenhouse (55%, **Figure 1B**). However, both PAR_HT_ and VPD_HT_ were elevated during milky HT, reaching approximately 327 µmol.m^-2^.s^-1^ and 0.7 kPa, respectively, compared with 230 µmol m^−2^ s^−1^ and 0.5 kPa in the open greenhouse (**Figures 1C, D**). This suggested that the climatic conditions before and after the milky HT period were similar between treatments. Meanwhile, during the milky HT period, the milky HT treatment experienced substantially higher T_HT_, PAR_HT_, and VPD_HT_, providing conditions sufficient to induce heat stress. Other environmental conditions were comparable between treatments.

### 3.2 The effect of milky-stage heat stress on rice phenology in relation to AGDD and GDD

**Figure 2:**
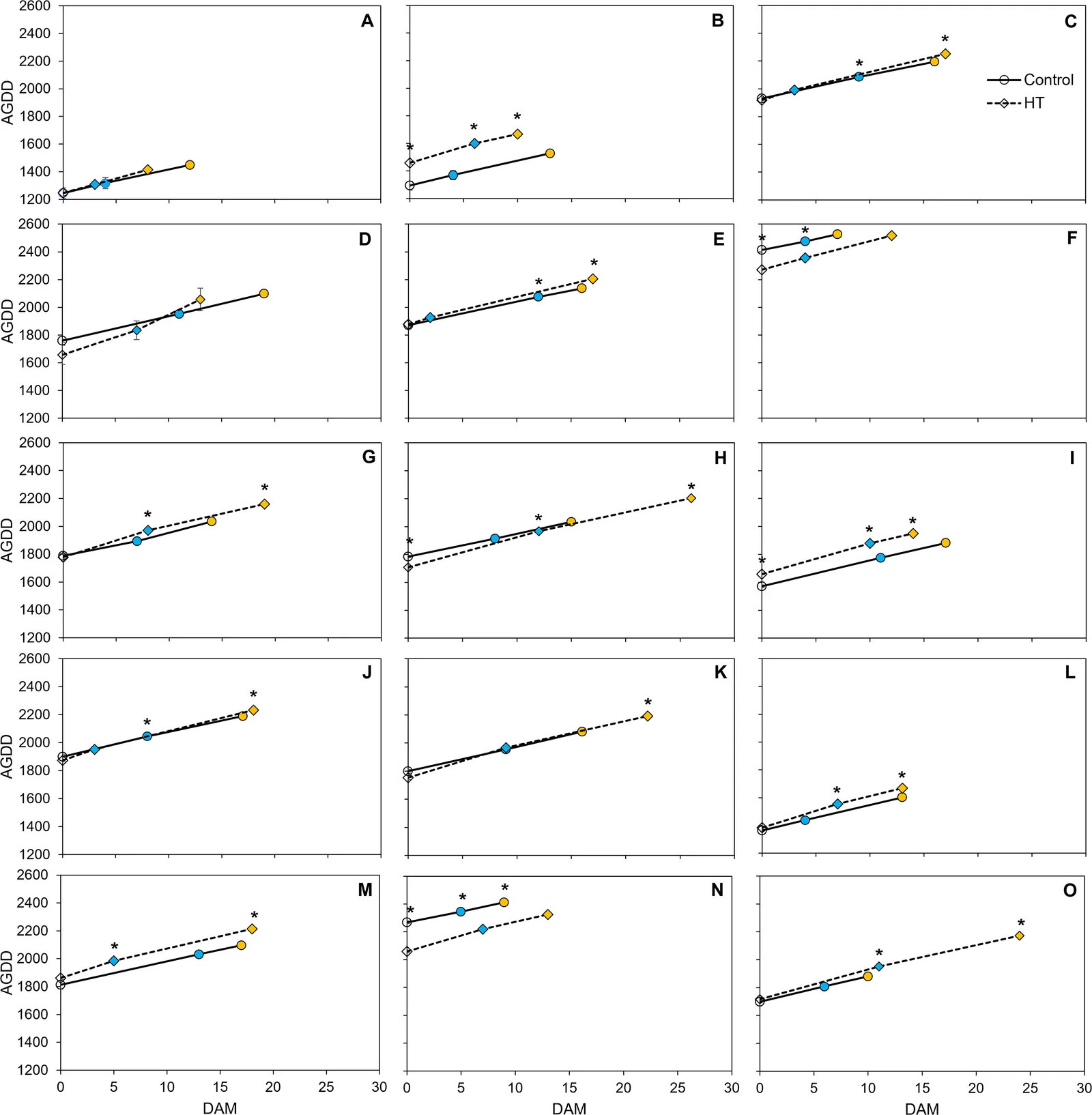
The effect of AGDD on phenology shift in different rice cultivars, namely, N22 **(A)**, Dular **(B)**, CN1 **(C)**, PSL2 **(D)**, PTT1 **(E)**, SPT1 **(F)**, RD29 **(G)**, RD31 **(H)**, RD41 **(I)**, RD49 **(J)**, RD57 **(K)**, RD61 **(L)**, RD63 **(M)**, Rice berry **(N)**, and IR64 **(O)** was investigated under both the control (open greenhouse) and milky HT conditions at days after the milky stage (DAM). Asterisk (*) indicates a significant difference at *p* ≤ 0.05 between the control and milky HT at the same growth stage (open circle = milky; blue circle =dough stage; and yellow circle = PM), as determined by an independent-samples *t*-test (means ± SE, n = 3–4).

Under the control (open greenhouse), the accumulated growing degree days (AGDD) from the milky stage to the physiological maturity (PM) stage showed a slight increase, except in SPT1 and Rice berry. However, AGDD during milky-stage HT sharply increased in certain rice cultivars, namely, Dular, RD41, and RD63, as shown in **Figure 2**. Additionally, AGDD in most other rice cultivars exhibited a slight increase due to milky HT. The developmental duration from the milky stage to the PM stage was shortened by approximately 4.5, 4.0, and 3.5 days in N22, Dular, and RD41, respectively, in samples undergoing milky-stage HT. However, in the remaining cultivars, the duration from the milky stage to PM was extended by 1 to 13.25 days, except for RD61, for which phenological changes were not statistically significant.

The prolonged phenology from the dough stage to the PM stage observed in all rice cultivars except N22, Dular, RD41, and RD61 was associated with higher AGDD. N22 experienced the highest degree of shortened phenology at M-PM due to a slight increase in AGDD under milky HT. In contrast, IR64 exhibited the greatest prolonged phenology under the same condition. N22 and RD41 showed shortened phenology during both the milky-to-dough and dough-to-PM stages, corresponding to an increase in AGDD due to milky HT. These findings suggest that the increase in AGDD (from the milky stage to PM compared with the control) due to milky HT contributed to the shortened phenology from the milky stage to PM in the heat-tolerant cultivar, such as N22, and in the non-classified heat-tolerant/sensitive cultivar, including RD41. Dular, also all cultivars exhibited shortened phenology from the dough stage to PM, leading to shortened phenological shift at milky-PM. In addition, the increase in AGDD (from the dough stage to PM compared with the control) due to milky HT was associated with a forward phenological shift at milky-PM in the majority of rice cultivars, except RD61.

**Figure 3:**
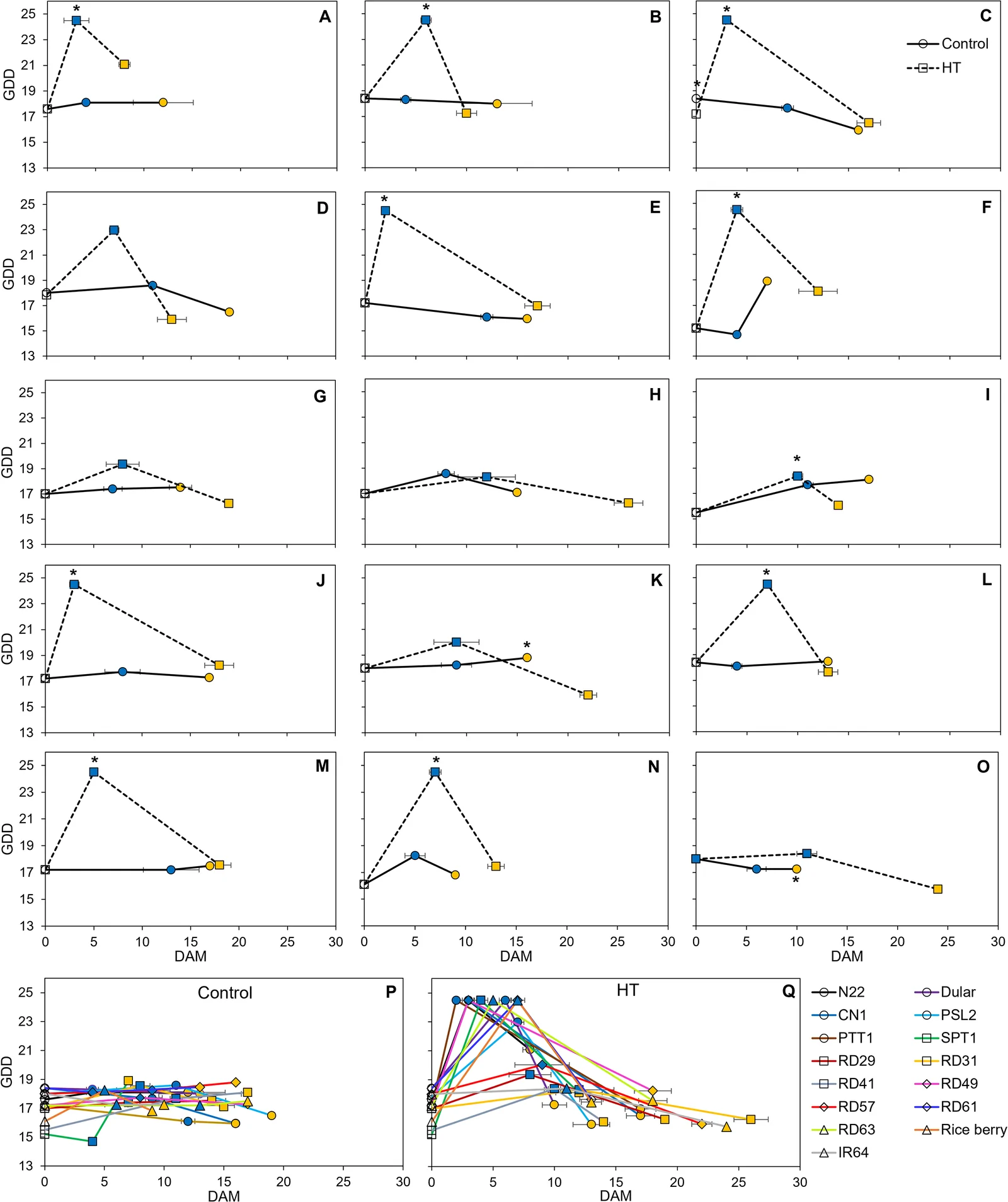
The effect of GDD on phenology shift in different rice cultivars, namely, N22 **(A)**, Dular **(B)**, CN1 **(C)**, PSL2 **(D)**, PTT1 **(E)**, SPT1 **(F)**, RD29 **(G)**, RD31 **(H)**, RD41 **(I)**, RD49 **(J)**, RD57 **(K)**, RD61 **(L)**, RD63 **(M)**, Rice berry **(N)**, and IR64 **(O)** was investigated under both the control (open greenhouse) and milky HT conditions at days after the milky (DAM) stage. P and Q represent the comparison among the 15 rice cultivars under both control and milky HT, respectively. Asterisk (*) indicates a significant difference at *p* ≤ 0.05 between the control and milky HT at the same growth stage (open circle = milky; blue circle =dough stage; and yellow circle = PM), as determined by an independent-samples *t*-test (means ± SE, n = 3–4).

The growing degree days (GDD) under both the open greenhouse condition and milky HT was observed at milky-PM, as shown in **Figure 3**. During milky HT, the GDD trend increased during the milky-to-dough stage but then decreased sharply during the dough-to-PM stage across all rice cultivars. Notably, under the open greenhouse condition, only SPT1 exhibited a significant increase in GDD during the dough-to-PM stage, while SPT1 also showed the lowest GDD during the milky-to-dough stage. Shortened phenology due to GDD under milky HT was observed in N22, Dular, and RD41, with N22 experiencing the highest degree of shortened phenology. In contrast to N22, IR64 exhibited the greatest prolonged phenology under the same condition. Thus, the increase in GDD (from the milky stage to PM compared with the control) due to milky HT contributed to a shortened phenology at milky-PM in heat-tolerant rice, such as N22, and in non-classified heat-tolerant/sensitive rice, including RD41. However, Dular exhibited shortened phenology from the dough stage to PM, causing shortened phenological shift at milky-PM. In contrast, the decrease in GDD (from the dough stage to PM compared with the control) due to milky HT induced the extended phenology shift at milky-PM in the majority of rice cultivars.

### 3.3 Effect of milky-stage heat stress on rice partitioning

**Figure 4:**
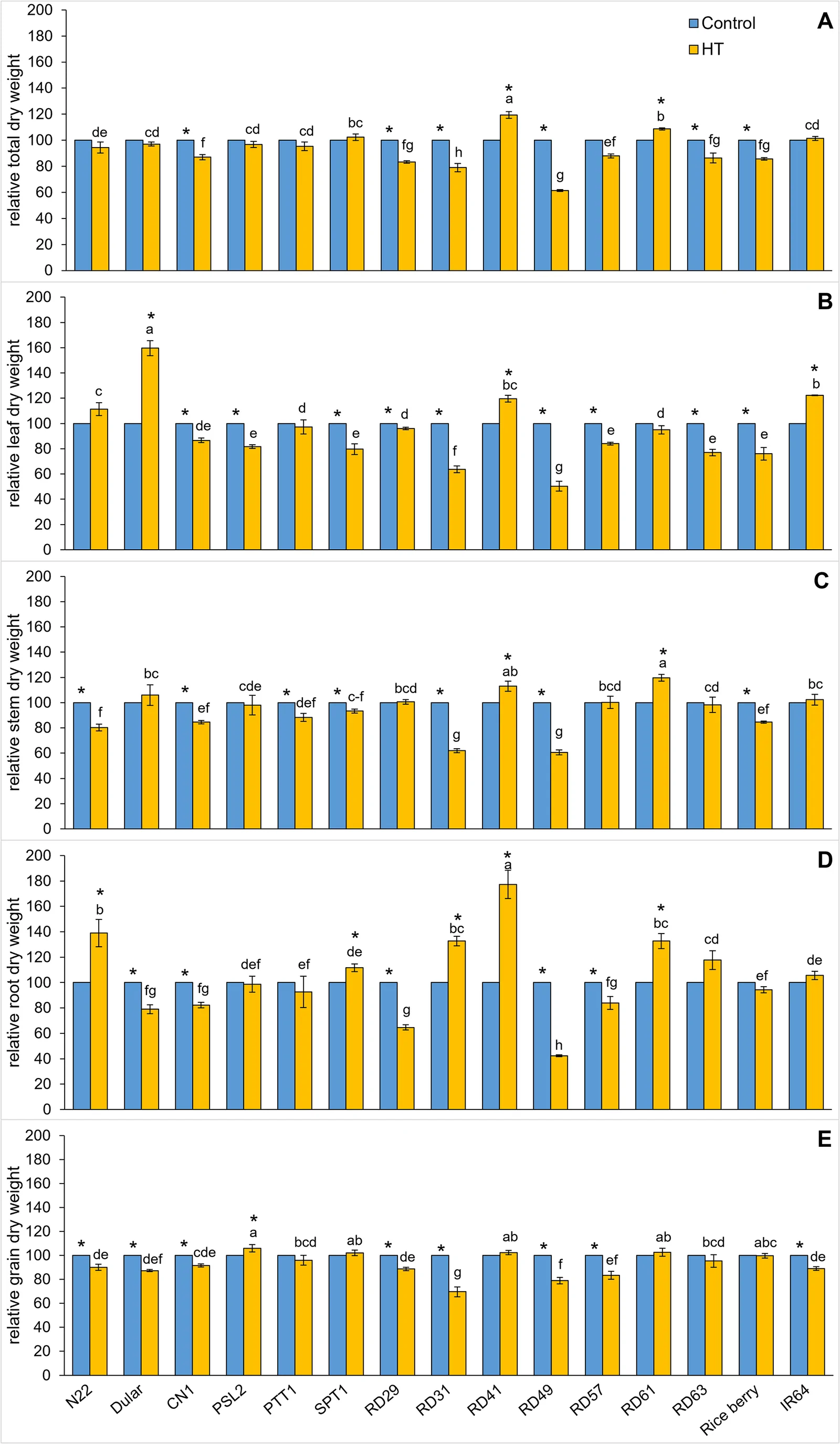
Partitioning of 15 rice cultivars presented in terms of relative total dry weight **(A)** and relative dry weight of the leaf **(B)**, stem **(C)**, root **(D)**, and grain **(E)**. The lowercase letters indicate significant differences among rice cultivars under the milky HT at p ≤ 0.05 by one-way ANOVA and DMRT (means ± SE, n = 3–4). The asterisk (*) indicates a significant difference between the control (blue) and milky HT (yellow) at p ≤ 0.05 by an independent-samples *t*-test.

The partitioning of control (open greenhouse) and milky HT across 15 rice cultivars are presented in terms of relative dry weight in **Figure 4**. Under milky HT, the highest increases in total dry weight (TDW), leaf dry weight (LDW), stem dry weight (STDW), root dry weight (RDW), and grain dry weight (GDW) were observed in RD41 (+19% relative to the control), Dular (+60% relative to the control), RD61 (+20% relative to the control), RD41 (+78% relative to the control), and PSL2 (+6% relative to the control), respectively.

In contrast to the overall increase in dry weight due to milky HT, the highest reductions in TDW, LDW, STDW, RDW, and GDW were observed in RD49 (-39% relative to the control), RD31 (-36% relative to the control), RD49 (-40% relative to the control), RD49 (-57% relative to the control), and RD31 (-37% relative to the control), respectively. Thus, these findings suggest that milky HT significantly enhanced dry weight accumulation in all plant parts of RD41, whereas RD49 experienced a rapid decline in dry weight across all organs. In addition, milky HT rapidly induced the translocation of assimilates to grains in PLS2. However, it inhibited or slowed assimilate translocation to grains in RD31 and RD49.

### 3.4 Effect of milky-stage heat stress on rice yield components and yield

Under milky HT, the yield components and yield of the 15 rice cultivars were affected, as shown in **Tables 2** and **3**. The greatest impact of milky HT on the number of grains per panicle (GPN), number of filled grains per panicle (FGPN), number of unfilled grains per panicle (UFGPN), 1000-grain weight (1000-GW), and seed set (SS) was observed in Rice berry (-12.12% relative to the control), RD63 (-17.72% relative to the control), CN1 (+333.33% relative to the control), Dular (-9.04% relative to the control), and CN1 (-14.79% relative to the control), respectively. The top four rice cultivars showing the highest reduction in each yield component due to milky HT were ranked as follows: 1) GPN: Rice berry < RD63 < RD29 < PTT1; 2) FGPN: RD63 < Rice berry < RD41 < RD29; 3) 1000-GW: Dular < IR64 < RD57 < RD49; and 4) SS: CN1 < PSL2 < RD63 < IR64. However, the top four largest increases in UFGPN due to milky HT were observed in CN1 > RD63 > RD61 > PSL2. Thus, these findings suggest that milky HT significantly affected RD63, leading to substantial reductions in yield components and yield.

**Table 2:** Yield components, including the number of grains per panicle (GPN) and the number of filled grains per panicle (FGPN), were determined under the control (open greenhouse) and the milky HT conditions (means ± SE, *n* = 3–4).

Rice cultivar	GPN			FGPN		
	Control	HT	% change	Control	HT	% change
N22	145 ± 3cd	143 ± 1c	-1.38	138 ± 3c	126 ± 2c*	-8.46
Dular	127 ± 3e	125 ± 1f	-1.83	114 ± 2ef	112 ± 1ef	-1.76
CN1	127 ± 5e	123 ± 3f	-3.66	107 ± 5fg	103 ± 2g	-4.03
PSL2	105 ± 4f	109 ± 2g	4.14	98 ± 2g	91 ± 2h	-7.79
PTT1	147 ± 8c	138 ± 4cd	-5.91	120 ± 5de	114 ± 5def	-5.00
SPT1	171 ± 4a	164 ± 2a	-3.90	160 ± 3a	148 ± 2a*	-7.48
RD29	145 ± 1cd	133 ± 1de*	-8.28	126 ± 2d	115 ± 1def*	-8.97
RD31	125 ± 3e	128 ± 2ef	1.87	108 ± 2f	108 ± 4fg	0.00
RD41	159 ± 6b	153 ± 3b	-3.77	150 ± 3b	134 ± 2b*	-10.25
RD49	143 ± 2cd	139 ± 1cd	-3.25	128 ± 1d	120 ± 1cd*	-6.27
RD57	135 ± 3cde	128 ± 2ef	-5.66	126 ± 6d	119 ± 2cde	-5.80
RD61	128 ± 0e	123 ± 1f*	-3.90	113 ± 4ef	109 ± 1fg	-3.54
RD63	137 ± 3cde	124 ± 2f*	-9.51	126 ± 2d	104 ± 4g*	-17.72
Rice berry	176 ± 2a	155 ± 2b*	-12.12	161 ± 2a	135 ± 2b*	-16.53
IR64	134 ± 2de	133 ± 2de	-0.75	123 ± 1de	116 ± 2def*	-5.71

Different lowercase letters within the same column indicate the significant differences at p ≤ 0.05 according to one-way ANOVA and DMRT. An asterisk (*) indicates a significant difference between control and milky HT at p ≤ 0.05 by an independent-samples *t*-test.

**Table 3:** The effect of milky HT on yield components and yield, including the number of unfilled grains per panicle (UFGPN), 1000-grain weight (1000-GW), and seed set (SS), were determined under the control (open greenhouse) and the milky HT conditions (means ± SE, *n* = 3–4).

Rice cultivar	UFGPN			1000-GW (g)			Seed set (%)		
	Control	HT	% change	Control	HT	% change	Control	HT	% change
N22	7.00 ± 1.00de	11.00 ± 1.00d	57.14	18.35 ± 0.07j	17.86 ± 0.17f	-2.67	93.99 ± 0.93abcd	92.18 ± 0.85a	-1.93
Dular	11.00 ± 2.00abc	12.00 ± 0.00cd	12.09	25.03 ± 0.42gh	22.76 ± 0.99e	-9.04	91.38 ± 1.11cdefg	90.23 ± 0.30abc	-1.26
CN1	6.00 ± 2.00e	26.00 ± 2.00aA	333.33	26.12 ± 0.31fg	29.77 ± 0.06abA	13.99	95.21 ± 1.76ab	81.13 ± 1.06fA	-14.79
PSL2	8.00 ± 2.00cde	17.00 ± 2.00bcA	112.50	30.52 ± 0.24b	31.10 ± 0.63a	1.92	92.65 ± 1.37abcd	83.93 ± 1.58efA	-9.41
PTT1	15.00 ± 1.00a	18.00 ± 1.00bA	20.00	26.04 ± 0.03fg	26.68 ± 0.38c	2.45	88.66 ± 0.30fg	86.88 ± 0.81cde	-2.02
SPT1	9.00 ± 1.00cde	17.00 ± 1.00bcA	88.89	24.27 ± 0.64h	27.33 ± 0.81cA	12.62	94.51 ± 0.31abc	90.26 ± 0.36abcA	-4.50
RD29	15.00 ± 2.00a	17.00 ± 1.00bc	15.53	28.60 ± 0.02cd	27.95 ± 0.06cA	-2.28	89.40 ± 1.49efg	86.65 ± 0.67cde	-3.08
RD31	15.00 ± 1.00a	16.00 ± 2.00bcd	6.67	27.66 ± 0.23de	28.31 ± 0.57bc	2.35	88.58 ± 0.65g	87.72 ± 1.568bcd	-0.97
RD41	11.00 ± 2.00bcd	20.00 ± 1.00bA	78.82	24.21 ± 0.40h	27.23 ± 0.50cA	12.49	93.68 ± 1.45abcd	87.82 ± 0.56bcdA	-6.26
RD49	13.00 ± 1.00abc	20.00 ± 2.00bA	51.31	29.19 ± 0.77c	27.41 ± 0.70c	-6.10	90.84 ± 0.43defg	85.83 ± 1.50deA	-5.51
RD57	14.00 ± 2.00ab	15.00 ± 2.00bcd	4.79	26.91 ± 0.49ef	24.91 ± 0.35dA	-7.43	89.73 ± 1.63efg	90.91 ± 1.69ab	1.32
RD61	6.00 ± 1.00e	16.00 ± 1.00bcdA	161.17	31.73 ± 0.42a	31.13 ± 0.35a	-1.89	94.88 ± 1.00ab	89.27 ± 0.83abcdA	-5.92
RD63	5.00 ± 1.00e	15.00 ± 3.00bcdA	200.00	21.08 ± 0.62i	22.50 ± 0.85e	6.70	95.97 ± 0.44a	87.47 ± 2.13bcdeA	-8.86
Rice berry	15.00 ± 0.00a	19.00 ± 1.00b	24.47	18.61 ± 0.28j	22.34 ± 0.12eA	20.04	91.07 ± 0.31defg	88.19 ± 0.70bcdA	-3.16
IR64	11.00 ± 1.00abc	18.00 ± 1.00b	63.64	26.54 ± 0.17ef	24.54 ± 0.54dA	-7.54	92.04 ± 0.24bcdef	86.25 ± 0.52deA	-6.29

Different lowercase letters within the same column indicate significant differences at p ≤ 0.05 according to one-way ANOVA and DMRT. The uppercase letter A indicates a significant difference between the open greenhouse and the milky HT treatments at p ≤ 0.05 by an independent-samples *t*-test.

### 3.5 Classification of rice cultivar responses to milky-stage heat stress

**Figure 5:**
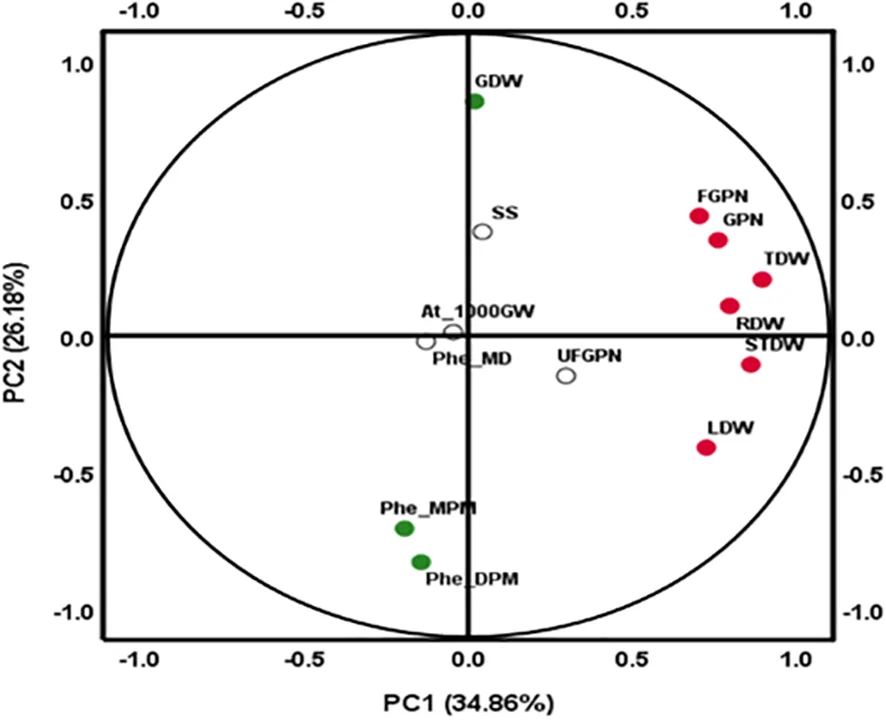
Principal component analysis (PCA) loading plot of phenology, dry weight, yield components, and yield of 15 rice cultivars under milky HT. Phe_MD, Phenology Milky-Dough; Phe_DPM, Phenology Dough-PM; Phe_MPM, Phenology Milky-PM; TDW, Total dry weight; LDW, Leaf dry weight; STDW, Stem dry weight; RDW, Root dry weight; GDW, Grain dry weight; GPN, number of grains per panicle; FGPN, number of filled grains per panicle; UFGPN, number of unfilled grains per panicle; 1000-GW, 1000-grain weight; SS, Seed set. Red circles, green circles, and open circles represent PC1, PC2, and PC3–PC4, respectively.

**Figure 6:**
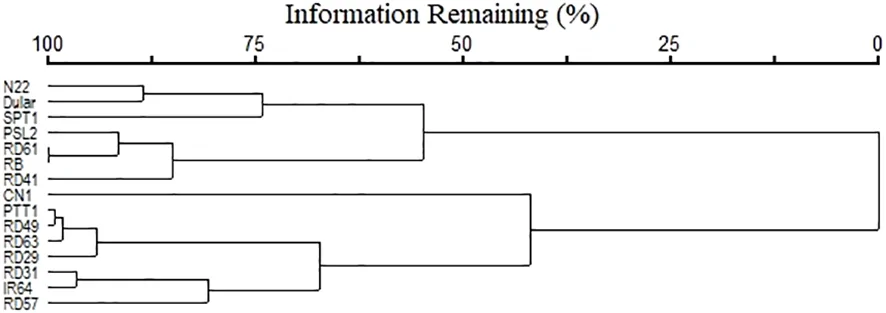
Dendrogram shows the classification of 15 rice cultivars using cluster analysis based on phenology at dough-PM, phenology at milky-PM, and seed set on 7 days after heat treatment at 41°C.

The 12 recommended rice cultivars, together with the standard heat-tolerant cultivars (N22 and Dular) and the heat-sensitive cultivar (IR64), were exposed to milky HT to classify their heat tolerance/sensitivity. This classification was based on an integrated approach that incorporated distinct differences between the standard heat-tolerant and heat-sensitive rice in each parameter, principal component analysis (PCA), Pearson’s correlation, and cluster analysis, as shown in **Figures 5** and **6** and **Tables 4** and **5**. The PCA was conducted to assess similarities and differences across 13 traits, including phenology, dry weight, yield components, and yield under milky HT (**Figure 5**). The analysis revealed a separation of 13 traits with PC1 = 34.86% and PC2 = 26.18%. Six traits (TDW, LDW, STDW, RDW, GDW, and FGPN) contributed to PC1, while three traits (Phe_DPM, Phe_MPM, and GDW) were associated with PC2. The six traits were identified as key indicators of high heat tolerance with high genetic variability in rice.

**Table 4:** Pearson’s correlation between phenology, dry weight, yield components, and yield of 15 rice cultivars under milky HT.

	Phe_MD	Phe_DPM	Phe_MPM	TDW	LDW	STDW	RDW	GDW	GPN	FGPN	UFGPN	GW_1000	SS
Phe_MD	1												
Phe_DPM	-0.011	1											
Phe_MPM	.594**	.798**	1										
TDW	-0.251	-0.208	-.319*	1									
LDW	-0.277	0.275	0.071	.636**	1								
STDW	-0.137	-0.051	-0.121	.802**	.640**	1							
RDW	-0.181	-0.245	-.309*	.782**	.523**	.654**	1						
GDW	-0.081	-.539**	-.498**	0.24	-0.237	-0.064	0.036	1					
GPN	-0.16	-.428**	-.450**	.616**	.314*	.476**	.498**	0.29	1				
FGPN	-0.078	-.509**	-.470**	.575**	0.206	.430**	.441**	.369*	.943**	1			
UFGPN	-0.031	0.246	0.188	.300*	.449**	0.211	0.255	-0.218	0.068	-0.037	1		
GW_1000	0.184	.343*	.393**	0.146	0.078	0.073	0.003	0.035	-.316*	-.339*	.488**	1	
SS	0.026	-.421**	-.338*	0.019	-.322*	-0.015	-0.043	.456**	.351*	.451**	-.803**	-.522**	1

Phe_MD, Phenology Milky-Dough; Phe_DPM, Phenology Dough-PM; Phe_MPM, Phenology Milky-PM; TDW, Total dry weight; LDW, Leaf dry weight; STDW, Stem dry weight; RDW, Root dry weight; GDW, Grain dry weight; GPN, number of grains per panicle; FGPN, number of filled grains per panicle; UFGPN, number of unfilled grains per panicle; 1000-GW, 1000-grain weight; SS, Seed set.

Asterisks (*, **) indicate significant difference by ANOVA at p ≤ 0.05 and 0.01, respectively.

**Table 5:** Phenology, dry weight, yield components and yield of fifteen rice cultivars for selection to classify heat tolerant/sensitive rice under milky HT by cluster analysis.

Parameters	Standard heat tolerant/sensitive rice			PCA	Pearson’s correlation	Parameters selected for dendrogram
	N22	Dular	IR64			
Phe_MD					✓	–
Phe_DPM		✓			✓	✓
Phe_MPM	✓	✓			✓	✓
TDW				✓	✓	Not suitable for distinguishing between heat-tolerant (N22, Dular) and heat-sensitive (IR64) cultivars
LDW				✓	✓	Not suitable for distinguishing between heat-tolerant (N22, Dular) and heat-sensitive (IR64) cultivars
STDW				✓	✓	Not suitable for distinguishing between heat-tolerant (N22, Dular) and heat-sensitive (IR64) cultivars
RDW				✓		–
GDW				✓		–
GPN					✓	–
FGPN				✓		–
UFGPN					✓	–
GW_1000					✓	–
SS			✓		✓	✓

Phe_MD, Phenology Milky-Dough; Phe_DPM, Phenology Dough-PM; Phe_MPM, Phenology Milky-PM; TDW, Total dry weight; LDW, Leaf dry weight; STDW, Stem dry weight; RDW, Root dry weight; GDW, Grain dry weight; GPN, number of grains per panicle; FGPN, number of filled grains per panicle; UFGPN, number of unfilled grains per panicle; 1000-GW, 1000-grain weight and SS, Seed set.

Thus, the considered three traits (Phe_DPM, Phe_MPM, and SS; **Table 5**) of 12 rice cultivars compared to N22, Dular, and IR64 were selected. Trait selection was based on a combination of PCA (six traits in PC1; **Figure 5**), significant Pearson’s correlation (Phe_MD, Phe_DPM, Phe_MPM, TDW, LDW, STDW, GPN, UFGPN, 1000-GW, and SS; **Table 4**), and the ability of the selected traits to distinguish between heat-tolerant and heat-sensitive cultivars (Phe_DPM, Phe_MPM, and SS). Using Phe_DPM, Phe_MPM, and SS traits for cluster analysis (**Figure 6**), the classification of heat tolerance and heat sensitivity under milky HT led to the identification of four groups: 1) heat tolerance (N22, Dular, and SPT1), 2) moderate heat tolerance (PSL2, RD61, Rice berry, and RD41), 3) moderate heat sensitive (CN1), and 4) heat sensitive (IR64, PTT1, RD29, RD31, RD49, RD57, and RD63).

## 4 Discussion

### 4.1 Milky-stage heat stress stimulates changes rice phenology

Rice phenology determines the duration of each growth stage and consequently influences yield formation. Exposure to temperatures above the threshold temperature (~35 °C) can accelerate crop development after anthesis and alter the duration of key growth stages, including panicle initiation, flowering, and maturity (Shi et al., 2015a; Sanwong et al., 2023; Chandarak et al., 2023). In the present study, the milky HT simulated a realistic heat stress condition, following a diurnal temperature cycle with a daily peak of 41 °C for 3 h. The daily mean daytime temperature during milky HT was 35.83 °C, which exceeded the maximum temperature observed in the open greenhouse (31.5 °C). This temperature regime was based on ambient air temperature records from field climate data collected during the summer season at the Agronomy Field Station, Khon Kaen University. Thus, the heat treatment simulated heat conditions usually experienced by rice during grain filling in tropical environments. In addition, simulated milky-stage heat stress increased AGDD and induced contrasting phenological responses among rice cultivars. Heat-tolerant cultivars, including N22, Dular, and RD41, exhibited shortened phenology from the milky to PM stages. In contrast, heat-sensitive cultivars, especially IR64 and several recommended cultivars, showed prolonged phenology under the same conditions. These findings indicate that phenological responses to milky HT are genotype-dependent and closely associated with heat tolerance. In addition, the response in N22, Dular, and RD41 may represent a heat-escape strategy, whereby plants complete grain filling more rapidly and reduce the duration of exposure to heat stress. In contrast to N22, Dular, and RD41, the response of IR64 may reflect heat-induced disruption of grain development and assimilate allocation, resulting in delayed maturation. This suggests that heat stress can either accelerate or delay crop development depending on genotype, thermal sensitivity, and source–sink dynamics. Therefore, the contrasting phenological responses observed in this study demonstrated that rice cultivars employ different adaptive strategies under heat stress, with tolerant cultivars maintaining developmental progression and sensitive cultivars experiencing delayed grain filling and maturity. In contrast, shortened phenology under high temperatures was reported in hybrid rice and the heat-sensitive cv. PTT1 (Rani and Maragatham, 2013; Sanwong et al., 2023). These differences may be related to the duration of heat stress and the growth stage at which heat stress occurred.

Growing degree days (GDD) play a crucial role in all growth stages of rice plants and generally increase as rice progresses through phenological stages during reproductive and maturity phases (Isalam and Sikder, 2011). In the present study, milky-stage heat stress altered GDD and phenological duration in a genotype-dependent manner. Increased GDD was associated with shortened phenology in heat-tolerant cultivars, whereas reduced GDD contributed to prolonged phenology in heat-sensitive cultivars. This is similar to previous findings in IR64 under booting-stage heat stress (Chandarak et al., 2023). As GDD is influenced by both temperature and growth, these findings suggest that both booting and milky HT alter phenological development through changes in thermal accumulation, depending on heat-tolerant cultivars (Chen and Gong, 2021). Thus, the shortened phenology observed in N22, Dular, and RD41 was accompanied by relatively stable assimilate partitioning and grain production, whereas the prolonged phenology of IR64 and PTT1 was associated with reduced assimilate translocation and poorer yield performance. This indicates that phenological responses were closely linked to source–sink regulation under heat stress.

### 4.2 Milky-stage heat stress alters assimilate translocation

Milky-stage heat stress altered dry matter partitioning and assimilate translocation among rice cultivars. During grain filling, flag leaves play a crucial role as a source for synthesizing starch and supplying assimilates to developing grains. Heat stress can impair photosynthetic performance, reduce starch synthesis, and disrupt assimilate transport, resulting in altered biomass allocation and reduced grain development in rice plants (Lü et al., 2013; Pansarakham et al., 2018). Previous studies showed that heat stress decreases photosynthetic rate and panicle dry weight, leading to reduced grain yield (Lü et al., 2013; Dubey et al., 2018). Therefore, the different partitioning responses among cultivars may affect differences in their ability to maintain source activity (assimilate production) and sink strength (grain filling) under heat stress. Rice cultivars with greater dry matter accumulation and grain filling may maintain photosynthetic carbon assimilation and assimilate translocation, whereas rice cultivars with reduced biomass accumulation experienced reduced assimilate transport to developing grains under milky-stage heat stress (Chandarak et al., 2023). However, in the present study, the average PAR inside the temperature chamber was 230 µmol m^−2^ s^−1^ compared with 392 µmol m^−2^ s^−1^ in the open greenhouse. The reduced irradiance inside the temperature chamber may have decreased photosynthetic carbon assimilation and subsequently influenced assimilate availability, dry matter partitioning, and grain filling. Therefore, some of the observed responses may reflect the combined effects of heat stress and reduced light intensity. Because all rice cultivars were exposed to the same environmental conditions, the relative comparisons among cultivars remain valid for evaluating heat-stress responses. Future studies integrating direct measurements of photosynthesis, assimilate translocation, and grain filling under field heat stress conditions or higher irradiance would help clarify the interactive effects of temperature and light on rice performance during the milky stage.

### 4.3 Milky-stage heat stress impacts yield components and yield

Rice yield is highly sensitive to heat stress, which can reduce both yield quantity and quality (Lyman et al., 2013). Previous studies reported that heat stress during reproductive and grain-filling stages, including booting, flowering, 50% flowering, and grain filling, significantly reduces rice yield (Bahuguna et al., 2017; Thuy and Saitoh, 2017; Xu et al., 2021). According to these findings, milky-stage heat stress reduced seed set (SS) and increased the number of unfilled grains per panicle (UFGPN) in sensitive cultivars. This response may be associated with heat stress-induced reductions in photosynthetic performance, crop growth, and assimilate production, leading to lower grain filling and yield (Chandarak et al., 2023). The milky stage is the beginning of the grain-filling stage, during which carbohydrates, proteins, and lipids are synthesized and transported into the seeds (Sreenivasulu et al., 2015). Consequently, heat stress during the grain-filling stage can impair endosperm and embryo development, reduce grain weight and yield, and alter grain quality traits, including amylose, protein, anthocyanin content, and invertase activity (Pravallika et al., 2020; Xu et al., 2021). Heat stress also alters starch composition through reductions in amylose content and changes in amylopectin structure during seed development (Inouchi et al., 2000). However, it should be noted that photosynthetic performance, carbohydrate metabolism, and enzyme activities were not measured in the present study. Therefore, the proposed mechanisms related to assimilate production, source–sink balance, grain filling, starch synthesis, and sink strength should be considered based on yield responses and previous reports rather than direct evidence from this experiment. Further physiological and biochemical studies are required to verify these mechanisms and clarify how milky-stage heat stress influences grain development and yield formation in rice.

In addition, heat stress can reduce pollen viability, restrict anther dehiscence, and inhibit pollen tube growth, resulting in lower seed set and grain weight (Shrestha et al., 2022; Zhang et al., 2023). Meanwhile, heat-tolerant cultivars are able to maintain pollen fertility and seed set under heat stress, preserving grain yield (Zafar et al., 2022). Surprisingly, an increase in 1000-grain weight was observed in CN1 under milky HT. This may be a compensatory mechanism involving reduced grain number, increased sink strength, and altered source–sink balance, contributing to increased grain weight. Additionally, milky HT may increase UFGPN by impairing grain filling and disrupting starch synthesis, leading to irregular starch granules and greater grain chalkiness (Ishimaru et al., 2020). Heat stress also alters starch composition through reductions in amylose content and changes in amylopectin structure during seed development (Inouchi et al., 2000). Therefore, while the present study provides insights into phenological responses, assimilate partitioning, and yield formation under milky-stage heat stress, it does not capture potential heat-induced changes in grain quality. Future studies should incorporate quality-related parameters, such as chalkiness score, amylose content, protein content, and starch composition, to achieve a more comprehensive assessment of rice heat tolerance and grain quality stability under heat conditions.

### 4.4 The variation in the response of heat-tolerant and heat-sensitive rice exposed to milky-stage heat stress

Recent studies have highlighted the importance of phenological plasticity, assimilate allocation, grain filling, and reproductive stability as key determinations of rice performance under reproductive-stage heat stress (Xu et al., 2021; Shrestha et al., 2022; Chandarak et al., 2023; Kumar et al., 2025). Our findings support these recent advances and further demonstrate that cultivar-specific phenological and assimilate partitioning strategies contribute to heat tolerance during the milky stage, which has been less studied than flowering and late grain-filling stages. Based on phenological traits and seed set, 13 rice cultivars were classified into four groups: heat-tolerant, moderately heat-tolerant, moderately heat-sensitive, and heat-sensitive under milky HT. Heat-tolerant cultivars exhibited the ability to maintain assimilate allocation and reproductive performance under milky HT. Interestingly, different tolerance strategies were observed among tolerant cultivars. N22 and Dular showed shortened phenology, which may represent a heat-escape mechanism that reduces the duration of heat stress exposure. Meanwhile, SPT1 maintained grain filling through prolonged phenology. These responses indicate that heat tolerance during the milky stage is associated with the maintenance of assimilate translocation and grain development rather than a single phenological pattern. Similar responses have been reported in heat-tolerant rice cultivars that maintained photosynthetic performance and assimilate allocation under booting-stage heat stress (Chandarak et al., 2023). This suggests that N22 and Dular may employ a potential heat-escape mechanism through shortened phenology, while SPT1 exhibits tolerance via prolonged grain filling. Thus, the contrasting responses suggested that heat tolerance in rice arises from multiple mechanisms, such as phenological escape, maintenance of source–sink balance, or extended grain-filling capacity. As a result, N22, Dular, and SPT1 maintained grain filling and yield under milky HT, which contributed to their classification as heat-tolerant cultivars.

Moderately heat-tolerant cultivars maintained biomass accumulation and grain development under milky HT by sustaining assimilate allocation to vegetative and reproductive organs. Although their phenological responses differed, heat-tolerant cultivars were generally able to preserve dry matter accumulation and grain filling, resulting in only moderate reductions in seed set. In particular, higher 1000-grain weight compensated for reductions in other yield components. In the case of RD41, it showed several responses to milky HT, including shortened phenology and increased dry matter accumulation. This placed it between heat-tolerant and heat-sensitive groups. Particularly, its seed set and yield stability were lower than those of the standard heat-tolerant cultivars N22 and Dular. Thus, the combined evaluation based on phenological traits, seed set, PCA, Pearson’s correlation, and cluster analysis indicated that RD41 is moderately heat-tolerant rather than fully heat-tolerant. In contrast, moderately heat-sensitive and heat-sensitive cultivars exhibited prolonged phenology but reduced biomass accumulation and assimilate allocation to developing grains. These responses resulted in lower filled grain number and seed set, indicating that prolonged phenology alone was insufficient to maintain grain filling and yield under heat stress. However, CN1 showed a higher 1000-grain weight than other heat-sensitive cultivars, supporting its classification as moderately heat-sensitive.

## 5 Conclusion

This study concluded that rice cultivars showed different phenology, assimilate partitioning, and yield responses to milky HT. The integrated analyses identified phenological duration from milky to PM, dough to PM, and seed set as key traits associated with heat tolerance. Based on these traits, rice cultivars were classified into four groups: heat-tolerant (N22, Dular, and SPT1), moderately heat-tolerant (PSL2, RD61, Rice berry, and RD41), moderately heat-sensitive (CN1), and heat-sensitive (IR64, PTT1, RD29, RD31, RD49, RD57, and RD63). Overall, the milky stage is a critical period of early grain filling, when endosperm cells develop and accumulate in starch reserves. During this period, developing grains act as major sinks and require continuous assimilate translocation from photosynthetically active sources, especially leaves and stems. Milky-stage heat stress can disrupt grain development, shorten grain-filling period, and disrupt source–sink relationship, reducing assimilate translocation to grains and reducing grain weight and yield. Thus, alteration of dry matter partitioning, grain dry weight, and yield components was observed in this study, reflecting heat-disrupted assimilate allocation during grain filling. The relationship between grain dry weight and yield in heat-tolerant rice cultivars indicates high potential to maintain assimilate translocation and sink activity under heat stress. Meanwhile, heat-sensitive cultivars showed greater reductions in these traits. In addition, heat tolerance strategies may involve either a heat-escape mechanism via shortened phenology to avoid heat exposure or prolonged phenology to extend grain filling. The shortened phenology was observed only in some tolerant cultivars (N22, Dular, RD41) and is not the sole indicator of heat tolerance. Nevertheless, further studies across multiple growing seasons and environments are required to confirm the stability of these heat-tolerant cultivars and their relevance for heat-tolerant rice breeding programs.

## Data Availability

The raw data supporting the conclusions of this article will be made available by the authors, without undue reservation.
