# Correction: Heat stress during the milky stage reshapes phenology, assimilate partitioning, and yield formation in rice cultivars with contrasting heat tolerance

**DOI:** 10.3389/fpls.2026.1936553

**Published:** 2026-08-13

**Authors:** Payu Pansarakham, Netnapa Sumtonglang, Panupon Hongpakdee, Charanya Kulya, Piyada Theerakulpisut, Anoma Dongsansuk

**Affiliations:** 1 Research Center and Central Laboratory, Faculty of Agriculture, Khon Kaen University, Khon Kaen, Thailand; 2 Department of Agronomy, Faculty of Agriculture, Khon Kaen University, Khon Kaen, Thailand; 3 Salt-Tolerant Rice Research Group, Khon Kaen University, Khon Kaen, Thailand; 4 Department of Horticulture, Faculty of Agriculture, Khon Kaen University, Khon Kaen, Thailand; 5 Department of Science and Technology, Faculty of Liberal Arts and Science, Roi-Et Rajabhat University, Roi Et, Thailand; 6 Department of Biology, Faculty of Science, Khon Kaen University, Khon Kaen, Thailand

**Keywords:** heat stress, phenology, photo-assimilate translocation, rice, yield

The citation for “Mahmood et al., 2021” has been removed.

The publisher location for “Daubenmire, 1974” was missing. It should be “Daubenmire, R. F. (1974). *Plants and environment. 3rd ed*. (New York, NY: Wiley), 1–432.”.

The reference details for “Ashish et al. (2017)” were missing. They should be “Ashish, K. C., Krishnan, P., and Ramakrishnan, B. (2017). High temperature effects on rice growth, yield, and grain quality. *Adv. Agron*. 111, 87–206. doi: 10.1016/bs.agron.2017.03.011”.

In the **Acknowledgements,** “and Mr. Payu Pansarakham, who helped us analyze data and prepare the contents in this research” has been removed.

There was a mistake in **Table 5** as published. In rows “LDW” and “STDW”, column “Parameters selected for dendrogram”, it should read “Not suitable for distinguishing between heat-tolerant (N22, Dular) and heat-sensitive (IR64) cultivars”. The corrected **Table 5** appears below.

**Table 5:** Phenology, dry weight, yield components and yield of fifteen rice cultivars for selection to classify heat tolerant/sensitive rice under milky HT by cluster analysis.

Parameters	Standard heat tolerant/sensitive rice			PCA	Pearson ‘s correlation	Parameters selected for dendrogram
	N22	Dular	IR64			
Phe_MD					✓	–
Phe_DPM		✓			✓	✓
Phe_MPM	✓	✓			✓	✓
TDW				✓	✓	Not suitable for distinguishing between heat-tolerant (N22, Dular) and heat-sensitive (IR64) cultivars
LDW				✓	✓	Not suitable for distinguishing between heat-tolerant (N22, Dular) and heat-sensitive (IR64) cultivars
STDW				✓	✓	Not suitable for distinguishing between heat-tolerant (N22, Dular) and heat-sensitive (IR64) cultivars
RDW				✓		–
GDW				✓		–
GPN					✓	–
FGPN				✓		–
UFGPN					✓	–
GW_1000					✓	–
SS			✓		✓	✓

Phe_MD, Phenology Milky-Dough; Phe_DPM, Phenology Dough-PM; Phe_MPM, Phenology Milky-PM; TDW, Total dry weight; LDW, Leaf dry weight; STDW, Stem dry weight; RDW, Root dry weight; GDW, Grain dry weight; GPN, number of grains per panicle; FGPN, number of filled grains per panicle; UFGPN, number of unfilled grains per panicle; 1000-GW, 1000-grain weight and SS, Seed set.

The original version of this article has been updated.

