# Supplementary material for: Heat stress during the milky stage reshapes phenology, assimilate partitioning, and yield formation in rice cultivars with contrasting heat tolerance
Source: Front Plant Sci. 2026 Jul 9;17:1875326. doi: 10.3389/fpls.2026.1875326 (PMC13391291; doi:10.3389/fpls.2026.1875326)
Supplement: Supplementary file 1 [file Table1.docx]

**Table S1** The actual date in each growth stage and growth duration of 15-rice cultivars before milky-stage heat stress treatment

| No. | Rice cultivars | Transplanting  date | Flowering stage  date | Milky stage  date | Transplanting  (days) | seedling stage (days) | Panicle initiation stage (days) | Flowering stage  (days) | Milky stage  (days) |
| --- | --- | --- | --- | --- | --- | --- | --- | --- | --- |
| 1 | N22 | 1 Jun. 2018 | 26 Jul. 2018 | 29 Jul. 2018 | 7 | 28 | 36.00 | 65.00 | 67.00 |
| 2 | Dular | 29 May. 2018 | 26 Jul. 2018 | 29 Jul. 2018 | 7 | 28 | 41.00 | 67.75 | 69.75 |
| 3 | CN1 | 26 Apr. 2018 | 25 Jul. 2018 | 29 Jul. 2018 | 7 | 28 | 70.00 | 101.00 | 103.50 |
| 4 | PSL2 | 5 May. 2018 | 26 Jul. 2018 | 29 Jul. 2018 | 7 | 28 | 61.00 | 91.75 | 94.00 |
| 5 | PTT1 | 30 Apr. 2018 | 24 Jul. 2018 | 29 Jul. 2018 | 7 | 28 | 66.00 | 97.00 | 100.50 |
| 6 | SPT1 | 30 May. 2018 | 26 Jul. 2018 | 29 Jul. 2018 | 7 | 28 | 75.00 | 128.00 | 130.00 |
| 7 | RD29 | 3 May. 2018 | 25 Jul. 2018 | 29 Jul. 2018 | 7 | 28 | 39.00 | 94.25 | 97.25 |
| 8 | RD31 | 2 May. 2018 | 26 Jul. 2018 | 29 Jul. 2018 | 7 | 28 | 58.00 | 94.75 | 97.00 |
| 9 | RD41 | 16 May. 2018 | 25 Jul. 2018 | 29 Jul. 2018 | 7 | 28 | 45.00 | 81.00 | 83.75 |
| 10 | RD49 | 28 Apr. 2018 | 25 Jul. 2018 | 29 Jul. 2018 | 7 | 28 | 56.00 | 99.25 | 102.00 |
| 11 | RD57 | 3 May. 2018 | 26 Jul. 2018 | 29 Jul. 2018 | 7 | 28 | 60.00 | 94.25 | 96.25 |
| 12 | RD61 | 26 May. 2018 | 25 Jul. 2018 | 29 Jul. 2018 | 7 | 28 | 36.00 | 71.00 | 73.75 |
| 13 | RD63 | 2 May. 2018 | 25 Jul. 2018 | 29 Jul. 2018 | 7 | 28 | 60.00 | 95.00 | 97.50 |
| 14 | Riceberry | 6 Apr. 2018 | 25 Jul. 2018 | 29 Jul. 2018 | 7 | 28 | 70.00 | 120.75 | 123.50 |
| 15 | IR64 | 9 May. 2018 | 25 Jul. 2018 | 29 Jul. 2018 | 7 | 28 | 45.00 | 88.25 | 90.75 |

The milky stages are determined according to the growth stage of each rice cultivar.
