# Supplementary material for: Heat stress during the milky stage reshapes phenology, assimilate partitioning, and yield formation in rice cultivars with contrasting heat tolerance
Source: Front Plant Sci. 2026 Jul 9;17:1875326. doi: 10.3389/fpls.2026.1875326 (PMC13391291; doi:10.3389/fpls.2026.1875326)
Supplement: Supplementary file 2 [file Table2.docx]

**Table S2** Partitioning of fifteen rice cultivars present in term of actual total DW, leaf DW, stem DW, root DW and grain DW.

| No. | Rice cv. | Total DW (g) | | Leaf DW (g) | | Stem DW (g) | | Root DW (g) | | Grain DW (g) | |
| --- | --- | --- | --- | --- | --- | --- | --- | --- | --- | --- | --- |
|  |  | Control | HT | Control | HT | Control | HT | Control | HT | Control | HT |
| 1 | N22 | 110.64 | 104.25de | 12.87 | 14.35c | 33.51* | 26.92f | 14.23 | 19.80b* | 53.03* | 47.72de |
| 2 | Dular | 106.67 | 103.49ce | 8.92 | 14.24a* | 28.72 | 30.47bc | 20.48* | 16.25fg | 46.11* | 40.26def |
| 3 | CN1 | 151.10* | 131.36f | 27.02* | 23.39de | 46.94 | 39.89ef* | 32.71* | 26.92fg | 39.87 | 36.52cde* |
| 4 | PSL2 | 106.72 | 103.16cd | 21.41* | 17.53e | 40.28 | 39.39cde | 17.16 | 16.96def | 30.92 | 32.81a* |
| 5 | PTT1 | 137.84 | 131.54cd | 25.45 | 24.85d | 48.03* | 42.44def | 19.86 | 18.41ef | 44.87 | 43.07bcd |
| 6 | SPT1 | 159.03 | 162.47bc | 28.04* | 22.40e | 62.87* | 58.81c-f | 27.45 | 30.70de* | 43.61 | 44.37ab |
| 7 | RD29 | 160.21* | 133.58fg | 22.57* | 21.71d | 47.92 | 48.21bcd | 32.60* | 21.11g | 48.85* | 43.46de |
| 8 | RD31 | 134.24* | 106.04h | 24.34* | 15.52f | 53.12* | 32.76g | 16.65 | 22.10bc* | 50.99* | 35.46g |
| 9 | RD41 | 103.17* | 123.12a | 15.33 | 18.36bc* | 35.03 | 39.54ab* | 11.16 | 19.81a* | 46.35 | 47.35ab |
| 10 | RD49 | 163.15* | 100.04g | 30.93* | 15.54g | 54.27* | 32.80g | 26.80* | 11.40h | 45.90* | 36.12f |
| 11 | RD57 | 130.18 | 114.88ef | 21.73 | 18.24e* | 41.87 | 41.97bcd | 19.09* | 16.05fg | 51.03* | 42.54ef |
| 12 | RD61 | 102.19* | 111.19b | 12.61* | 11.93d | 27.76 | 33.25a* | 10.43 | 13.83bc* | 51.40 | 52.76ab |
| 13 | RD63 | 117.36* | 101.38fg | 24.60* | 18.95e | 43.92 | 43.08cd | 13.43 | 15.78cd | 34.67 | 33.13bcd |
| 14 | Rice berry | 167.61* | 143.98fg | 29.01* | 22.10e | 71.49* | 60.48ef | 29.91 | 28.25ef | 35.31 | 35.22abc |
| 15 | IR64 | 95.22 | 96.47cd | 15.76 | 19.26b* | 30.27 | 30.95bc* | 11.18 | 11.78de | 38.93* | 34.58de |

The small letters indicate significant difference among rice cultivars under the milky HT at p ≤ 0.05 by One Way ANOVA and DMRT (means ± SE, n = 3-4). The asterisk (*) shows a significant difference between control (blue) and milky HT (yellow) at p ≤ 0.05 by independent-samples *t*-test.
